# CAR T cells and T cells phenotype and function are impacted by glucocorticoid exposure with different magnitude

**DOI:** 10.1186/s12967-024-05063-4

**Published:** 2024-03-12

**Authors:** Thomas Poiret, Sara Vikberg, Esther Schoutrop, Jonas Mattsson, Isabelle Magalhaes

**Affiliations:** 1 Department of Oncology-Pathology, Karolinska Institutet, Stockholm, Sweden; 2 Gloria and Seymour Epstein Chair in Cell Therapy and Transplantation, Princess Margaret Cancer Centre and University of Toronto; Princess Margaret Cancer Centre, University Health Network, Toronto, ON Canada; 3 Department of Immunology and Transfusion Medicine, Karolinska University Hospital, Stockholm, Sweden

**Keywords:** Glucocorticoid, CAR, Chimeric antigen receptor, CD19, Mesothelin, 4-1BB, CD28

## Abstract

**Background:**

Chimeric antigen receptor (CAR) T cell therapy is associated with high risk of adverse events. Glucocorticoids (GCs) are cornerstone in the management of high-grade cytokine release syndrome (CRS) and immune effector cell-associated neurotoxicity syndrome (ICANS). Given the potentially deleterious effects of GCs on CAR T cells anti-tumor activity, increasing our understanding of GCs impact on CAR T cells is crucial.

**Methods:**

Using several CAR T cells i.e., CD19, mesothelin (MSLN)-CD28 and MSLN-41BB CAR T cells (M28z and MBBz), we compared phenotypical, functional, changes and anti-tumor activity between i) transduced CD19 CAR T cells with untransduced T cells, ii) M28z with MBBz CAR T cells induced by Dexamethasone (Dx) or Methylprednisolone (MP) exposures.

**Results:**

Higher levels of GC receptor were found in less differentiated CAR T cells. Overall, Dx and MP showed a similar impact on CAR T cells. Compared to untreated condition, GCs exposure increased the expression of PD-1 and TIM-3 and reduced the expression of LAG3 and function of T cells and CAR T cells. GC exposures induced more exhausted (LAG3 + PD1 + TIM3 +) and dysfunctional (CD107a-INFγ-TNF-IL2-) untransduced T cells in comparison to CD19 CAR T cells. GC exposure impaired more CD4 + than CD8 + CD19 CAR T cells. GC exposures increased more PD-1 expression associated with reduced proliferative capacity and function of M28z as compared to MBBz CAR T cells. CAR T cells anti-tumor activity was greatly affected by repeated GC exposure but partly recovered within 48h after GCs withdrawal.

**Conclusions:**

In summary, GCs impacted phenotype and function of untransduced and CAR T cell with different magnitude. The nature of the CAR costimulatory domain influenced the magnitude of CAR T cell response to GCs.

**Supplementary Information:**

The online version contains supplementary material available at 10.1186/s12967-024-05063-4.

## Background

Clinical trials and FDA/EMA approval in immunotherapy, especially cell-based therapy to treat hematological and solid malignancies, have drastically increased over the last decade. Some of these promising treatments such as CD19 CAR T cells treatments are now part of regular treatment options for refractory patients with B-acute lymphoblastic leukemia (ALL), diffuse large B cell lymphoma, mantle cell lymphoma, or follicular lymphoma. Despite this therapeutic revolution, many unmet needs remain, including prevention/reduction of treatment-related cytotoxicities namely, cytokine release syndrome (CRS) and immune effector cell-associated neurotoxicity syndrome (ICANS) [1]. For instance, in patients will ALL treated with CAR T cells, severe grade of CRS and ICANS (≥ grade 3) are often reported (incidence up to 71% and 56%respectively) [2]. CRS is characterized by an excessive immune activation and subsequent production (by CAR T cells but also macrophages) of pro-inflammatory cytokines (*e.g.* IL-6) [3]. Tocilizumab (anti-IL-6 receptor) is the most commonly used drug to treat high grade CRS, however, Tocilizumab has no central nervous system penetration which limits its efficacy in preventing or treating ICANS. Glucocorticoids (GCs), the other cornerstone of CRS/ICANS management, are used to treat patients with grade ≥ 2 CRS, patients with resistance to Tocilizumab and/or experiencing ICANS [4]. In patients with ALL, early co-administration of corticosteroids and Tocilizumab to prevent severe CRS does not appear to impact CD19 CAR T cell efficacy [5]. On the other hand, prolonged course (> 10 days) and higher cumulative dose of corticosteroids have been associated with shorter progression-free survival and a negative prognostic indicator for overall survival of patients treated with CD19 CAR T cells [6, 7]. While one clinical trial (ZUMA-1) has demonstrated that prophylactic GC for CD19 CAR therapy prevented high grade CRS and ICANS, the long-term effect of GC on CAR T cell durable response remains to be assessed [8].

GCs mediate their effect via the glucocorticoid receptor (GR) that regulates several physiological processes. GCs are mainly recognized as a powerful and universal immunosuppressant but have demonstrated various effects on T cells depending on differentiation or activation stage [9, 10]. The advent of commercial CD19 CAR T cell products, and the increasing number of patients treated with CAR T cells who may receive GCs, warrants a better understanding of the impact of immunosuppressive GCs on CAR T cells effector functions, expansion, and persistence. To date, the knowledge of GCs’ impact on CAR T cells comes essentially from retrospective analysis of treated patients [11].

In this study, we assessed*, *in vitro, the effect of a single and repeated exposures of two commonly used GCs: Dexamethasone (Dx) and Methylprednisolone (MP) on the phenotype, functionality and killing of untransduced T cells and CD19 CAR T cells. As CD28-CAR T cells have been described to induce higher frequency and more severe CRS and ICANS than 4-1BB-CAR T cells [12], we also investigated the GC impact on mesothelin (MSLN)-CD28 and MSLN-41BB CAR T cells (M28z and MBBz). This in vitro analysis of CAR T cells exposed to GCs aims to contribute to the understanding of the impact of widely commonly used drugs in the new era of immunotherapy.

## Materials and methods

### Samples

All blood samples were obtained from healthy volunteer buffy coats (Karolinska University Hospital, Huddinge, Sweden).

### Drugs

Methylprednisolone (MP, Sigma-Aldrich M3781) and Dexamethasone (Dx, Sigma-Aldrich D4902) were reconstituted in distilled water or in ethanol, respectively. The GCs were aliquoted and stored in – 20 °C freezer upon use.

### CAR T cell production

Peripheral blood mononuclear cells (PBMCs) were isolated from healthy volunteer buffy coats. T cell transduction was performed as described previously with γ-retroviral vectors encoding CD28-CD19 CAR (generously donated by Prof. S. Rosenberg, National Cancer Institute, Bethesda, USA), and M28z or MBBz (kindly provided by Prof. M. Sadelain, Memorial Sloan Kettering Cancer Center, New York, USA) [13]. Briefly, PBMCs were cultured in AIM-V medium (Invitrogen, ThermoFisher Scientific) supplemented with 5% human AB serum and 300 IU/mL of IL-2 (Proleukin; Novartis) incubated at 37 °C 5% CO_2_. Two days after T cell activation with anti-CD3 monoclonal antibody (50 ng/mL, OKT3; Biolegend) of the PBMCs at 10^6^/ml, transduction was performed using the spinoculation method in 24w-non-tissue culture plates coated with RetroNectin (Takara) as described previously (29,315,094). CAR T cells were further expanded up to 2 weeks before cryopreservation until use. Transduction results in a T cell product (CD3 median frequency > 95%) composed of a mix of transduced CAR T cells and untransduced (UT) T cells. Additional CAR T cells and K562 tumor cells culture information can be found in Additional file 2.

### Cell viability following GCs exposure

K562 and CAR T cells were exposed to different concentrations of GC: 0, 0.01, 0.1, 1, 10, 100 µg/ml of Dx and MP for 3 days at 1mi/ml in 200µl in a 96-well culture plate. Cell viability upon exposure to different concentrations of GCs was assessed at 72h by APC-Annexin V (BD) and 7AAD (BD) staining in diluted Annexin V binding buffer (BD). Viability frequency was determined by AnnexinV-7AAD- cells.

### Experimental designs

Phenotype and function were evaluated by flow cytometry analysis after i) a single 24h GC exposure or ii) 3 repeated GC exposure over 6-days period with different GC concentration based in their pharmaceutical equivalence (0.1 µg/ml and 10 µg/ml Dx or 0.5µg/ml and 50 µg/ml MP [14], Fig. 2B). In the second design, difference in functionality were evaluated after 3 stimulation with irradiated CD19 + or MSLN + K562 target cells (1:1 Effector:Target, E:T ratio) every 3 days followed by a 24h exposure with low (0.1 µg/ml Dx or 0.5µg/ml MP) or high (10 µg/ml Dx or 50 µg/ml MP) GC doses. GC exposure was performed for 24h before the 3rd stimulation with target cells (as described in Fig. 3D). All experiments were performed at 1mi/ml cell concentration in 24-well culture plates. RU-486 (Mifepristone, Sigma M8046 [15]), a potent antagonist of the glucocorticoid receptor (GR) was used at a 10^–5^ M concentration as additional control in indicated assays.

### Killing assay

CAR T cells were exposed twice with different GCs (No GC or 10 µg/ml Dx or 50 µg/ml MP) at 2 days interval in a 24w plate at 1mi/ml cell concentration. Three days after second exposure CAR T cells were then co-incubated at 2:1 E:T ratio with MLSN + GFP + K562 with or without GCs exposure in AIM-V medium supplemented with 5% human serum without IL-2. Specific killing potency and immunophenotype were assessed by flow cytometry after 24h co-incubation (“Flow cytometry analysis” section in Additional file 2).

### Recovery assay

After 3 exposures with different GCs (No GC or 10 µg/ml Dx or 50 µg/ml MP) over a week period as described in “killing assay” section above, CAR T cells were transferred to a fresh GC-free medium containing AIM-V medium supplemented with 5% human serum and 300 IU/mL IL-2. Immunophenotype and specific killing potency were assessed after the 48h resting period in GC-free medium as described in “Killing assay” section above and in “Flow cytometry analysis” section in Additional file 2.

### Flow cytometry analysis

Several panels were used to assess i) the GC Receptor (GR) expression level in CAR T cells; ii) the CAR T cell phenotype after GC exposure and/or stimulation with target cells; iii) the CAR T cell function by intracellular staining; iv) the CAR T cell proliferation and v) the killing ability of CAR T cells under different GC conditions (for detail, see in Additional file 2).

To evaluate the percentage of killing as compared to the controls, i.e. CAR T cells non-exposed to GC, the following formula was used:

$$K=\left(1-\left(\frac{\frac{\mathrm{alive \,experimental \,K}562\mathrm{ GFP}+\mathrm{ count}}{\mathrm{alive \,experimental \,T cells \,count}}}{\frac{\mathrm{alive \,control K}562\mathrm{ GFP}+\mathrm{ count}}{\mathrm{alive \,control \,T cells \,count}}}\right)\right)*100.$$

### Statistical analysis

Data analysis was performed with GraphPad Prism software (GraphPad Software, San Diego, California USA). Student t test, Wilcoxon matched-pairs signed rank test or Friedman test with Dunn’s correction was used to compare paired samples of 2 or multiple groups. Unpaired samples were compared using Mann–Whitney test or Kruskal–Wallis test (two groups or multiple groups). The 2-way ANOVA with Sidak’s correction was used to do multiple comparisons between groups of samples and GC variable or CAR constructs. For normalization, the frequency difference between GC exposed and non-exposed paired samples was calculated and represented as delta, Δ. The threshold of significance was set at 0.05.

## Results

### Differentiation status impacted GC receptor expression level between CAR T cells and untransduced T cells.

**Fig. 1:**
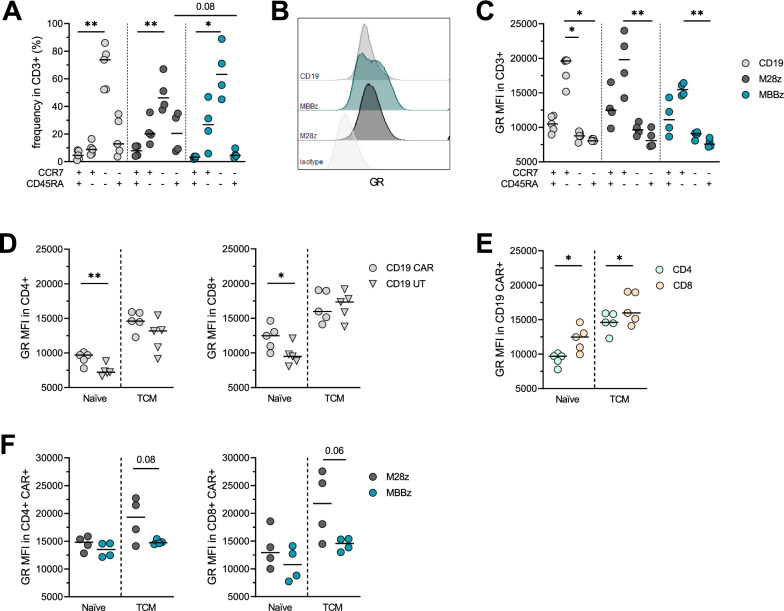
Phenotype of CD3 + T cells in CD19, M28z and MBBz CAR T cell products. **A** Differentiation subsets as defined by CD45RA and CCR7 expression **B** Representative histogram of glucocorticoid receptor (GR) staining in CD19, M28z and MBBz CAR T cell product in comparison with isotype control staining. **C** GR expression in CD3 + T cell subsets of CD19, M28z and MBBz CAR products. **D** GR expression in the naïve and central memory (TCM) subsets of CD19 CAR + and UT of CD4 + (left) and CD8 + (right) T cells. **E** Comparison of GR expression between CD4 + and CD8 + CD19 CAR T cells in the naïve and TCM memory subsets. **F** Comparison of GR expression between M28z and MBBz CAR T cells in the CD4 + (left) and CD8 + (right) naïve and TCM memory subsets. MFI: median fluorescence intensity. n = 5 donors for CD19 CAR T cells and n = 4 for M28z and MBBz CAR T cells. Friedman test was used to compare subsets within paired samples, Student t test was used to compare GR expression in different subsets. Medians are represented. *p < 0.05, **p < 0.01

CAR T cells, regardless of the CAR construct (CD19, M28z or MBBz CAR) presented a similar memory profile with a dominant effector memory phenotype (TEM, CCR7-CD45RA-, p < 0.05, Fig. 1A, Additional file 1: Figure S1A). No difference between CAR + and untransduced (UT) T cells was observed in the differentiation profile (Additional file 1: Figure S1B). We analyzed the GR expression level in CAR T cell subsets (Fig. 1B): The highest GR level was found in the central memory subset (CCR7 + CD45RA-, TCM) while and the lowest GR level in effector compartments (CCR7- CD45RA-/ + , TEM and TEMRA, Fig. 1C). CD19 + CAR CD4 + and CD8 + T cells exhibited a higher GR level than UT CD4 + and CD8 + T cells in the naïve subset (CCR7 + CD45RA +) (p < 0.01, Fig. 1D). This difference was not observed in TCM or other effector/memory subsets. Furthermore, CD8 + T cells showed a higher GR level than CD4 + T cells in the naïve and TCM CD19 CAR subsets (p < 0.05, Fig. 1E and Additional file 1: Figure S1C). Analysis of MSLN CARs encoding either the CD28 and 4-1BB co-stimulatory domains showed only a trend towards higher GR level in the TCM M28z CAR T cells (Fig. 1F and Additional file 1: Figure S1D).

While CAR T cells predominantly exhibited a TEM phenotype, the highest GR level was found in the TCM subset. Within the naïve and TCM subsets, higher GR levels were observed in CD8 + T cells and CD19 CAR T cells as compared to CD4 + and UT T cells, respectively. No significant difference in GR level was observed between M28z and MBBz CAR T cells. Although differences within some smaller, yet clinically relevant subsets (i.e. naïve and TCM) were observed, altogether the general GR expression levels were comparable between CAR and UT T cells.

### CAR T cells and UT T cells showed different phenotypical changes induced by GCs

**Fig. 2:**
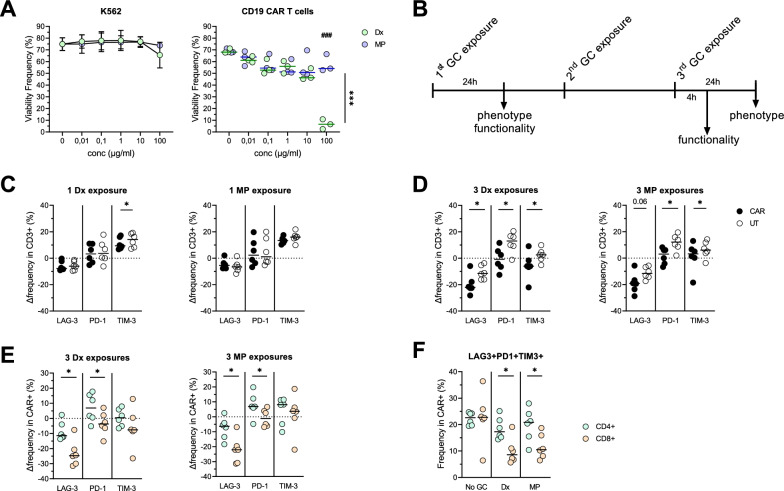
Impact of GCs exposure on untransduced (UT) and CD19 CAR + T cells phenotype. **A** Viability of K562 tumor cells (n = 3 replicates) and CAR T cells (n = 3 donors) after 72h of exposure with different concentration of Dexamethasone (Dx, green) or Methylprednisolone (MP, blue) determined by AnnexinV-7AAD-. 2-way ANOVA with Sidak’s correction was used to do multiple comparisons between viability of cells exposed to different Dx and MP concentration. **B** Experimental design for repeated GC exposures. Relative surface expression of LAG-3, PD-1 and TIM-3 in CAR + and UT T cells after one (1x, **C**) or 3 (3x, **D**) exposures with Dx (left) or MP (right). **E.** Relative expression of LAG-3, PD-1 and TIM-3 in CD4 + and CD8 + CAR + T cells after 3 exposures of Dx (left) or MP (right). **F** Comparison of LAG3 + PD1 + TIM3 + frequency between CD4 + and CD8 + CAR + T cells without GC or after 3 exposures with Dx or MP. **C-F**, n = 6 donors. Wilcoxon matched-pairs signed rank test was used to compare expressions in CAR + vs. UT or CD4 + vs. CD8 + T cells. Medians are represented. * p < 0.05, ***p < 0.001, ^###^p < 0.001

Following GR levels analysis, we aimed to investigate the phenotypical changes induced by GCs on CAR T cells. First, we evaluated the concentration window (0.01-100µg/ml) of Dx or MP that did not reduce drastically CAR T cell viability. Of note, the viability of CD19 + K562 tumor cells was not affected even at 100µg/ml of Dx or MP after 3 days (Fig. 2A). At the highest evaluated GCs concentration, reduced CAR T cell survival was observed, (p < 0.001), and CAR T cell viability was significantly more reduced by Dx than MP (p < 0.001, Fig. 2A). Following this viability screening, CD19 CAR T cells were exposed to 10 µg/ml Dx or 50 µg/ml MP (for GC dose equivalence) up to 3 times followed by phenotypical and functional assessment as described in Fig. 2B. Exposure (even repeatedly) of CD19 CAR T cells to GCs did not induce changes in CD19 CAR T cells frequency (median transduction rate of 42%) or CD4/CD8 ratio (Additional file 1: Figure S2A-B).

CD19 CAR and UT T cells phenotype was assessed following one and 3 repeated GC exposures. Overall, GCs induced a downregulation of LAG-3 and upregulation of PD-1 and TIM-3 (Additional file 1: Figure S2C-D). To compare the changes induced by the GC exposure between CD19 CAR T cells and UT T cells, impact of GC was assessed by looking at differences in frequency between GC exposed T cells and non-exposed T cells (Δ frequency): aside from increased TIM-3 expression in UT T cells upon Dx exposure (p < 0.05), single GC exposure did not show major differences between T cell fractions (Fig. 2C). On the other hand, repeated GC exposure induced a higher decrease in LAG-3 surface expression in CD19 CAR T cells (p < 0.05 Dx, and p = 0.06 MP) and a stronger increase in PD-1 and TIM-3 expressions in UT T cells (p < 0.05, Fig. 2D, Additional file 1: Figure S2E–F). The increased PD-1 expression induced by GC was reduced upon exposure with GC and the GR antagonist RU-486 confirming that GC is responsible for the phenotypical changes (Additional file 1: Figure S2G). No significant difference in CD19 CAR T cell phenotype was observed between Dx (10µg/ml) and MP (50µg/ml) exposure. Interestingly, when comparing changes in immune checkpoint markers (ICM) following GC exposure between CD4 + and CD8 + CD19 CAR T cells, LAG-3 was less downregulated and PD-1 more upregulated in CD4 + CAR T cells (p < 0.05, Fig. 2E). A similar trend was seen in UT T cells (Additional file 1: Figure S2H). Conversely, CD19 CAR CD4 + T cells exhibited a higher co-expression of LAG3 + PD1 + TIM3 + than CD19 CAR CD8 + T cells when exposed to GC (p < 0.05, Fig. 2F).

In our setting, repeated GC exposure impacted CD19 CAR and UT T cells to different extents as seen by a lesser exhausted phenotype in CD19 CAR T cells as compared to UT T cells. Additionally, within CAR T cells, CD4 + CAR T cells phenotype appeared more affected by GC exposure by retaining a higher triple expression of ICM.

### Upon GC exposure, CD19 CAR T cells retained better effector functions as compared to UT T cells.

In parallel to the phenotypic characterization, we assessed the impact of single and repeated GC exposure on the function of UT and CD19 CAR T cells.

**Fig. 3:**
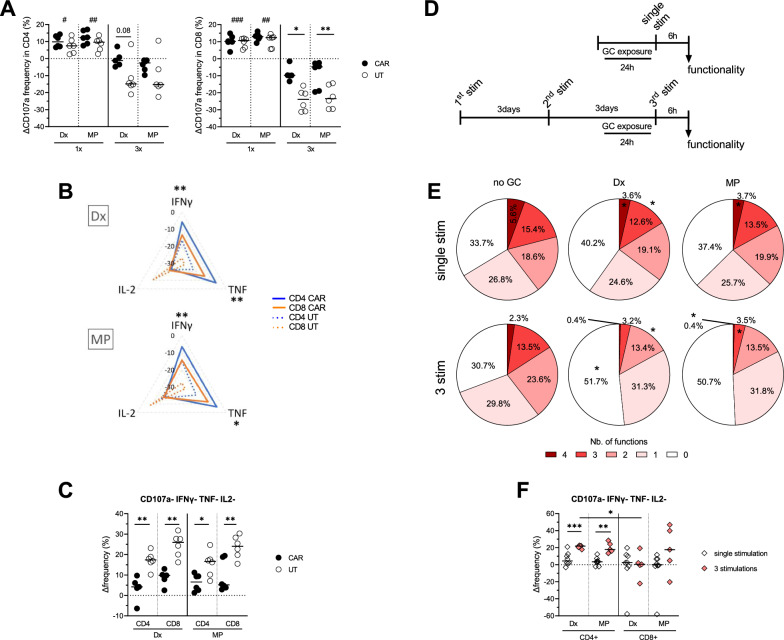
Impact of GCs exposure on CD19 CAR + T cells effector functions. **A** Relative frequency of CD107a + CD4 + (top) or CD8 + (bottom) CD19 CAR and UT T cells after one (1x) or 3 (3x) exposures with Dx or MP after PMA/ionomycin stimulation. **B** Radar plot of relative frequency of IFNγ + , TNF + or IL2 + CD4 + or CD8 + CD19 CAR and UT T cells after 3 exposures with Dx (top) or MP (bottom) after PMA/ionomycin stimulation. **C** Relative frequency of CD107a- IFNγ- TNF- IL2- CD4 + and CD8 + CAR and UT T cells after 3 exposures with Dx or MP after PMA/ionomycin stimulation. **D** Experimental design for single vs. repeated stimulation with K562 target cells with a single GC exposure. **E** Pie chart showing the number of functions of CD4 + CAR T cells without GC exposure compared to Dx or MP exposure following a single or 3 stimulations with CD19 + K562 tumor cells. **F** Comparison of the relative frequency of CD107a- IFNγ- TNF- IL2- CD4 and CD8 CD19 CAR T cells exposed to Dx or MP following a single and 3 stimulations with CD19 + K562 tumor cells. **A–C** n = 6 and **E–F** single stimulation n = 8 and 3 stimulation n = 5 donors. 2-way ANOVA with Sidak’s correction was used to compare CD107a + cells frequency at different doses of Dx and MP. Mann–Whitney test was used to compare the functions in CAR vs. UT or 1 × vs 3x. Friedman test with Dunn’s correction was used to compare multifunction in No GC vs. Dx vs. MP conditions. Student t test was used to compare CD107a expression between 1 × vs. 3x (#) and CD107a- IFNγ- TNF- IL2- CD4 and CD8 relative frequency between CD4 + and CD8 + T cells. Medians are represented. * p < 0.05, ** p < 0.01, *** p < 0.001. ^#^p < 0.05, ^##^p < 0.01, ^###^ p < 0.001

Upon GC exposure, both UT and CD19 CAR T cells were stimulated using PMA and ionomycin. A single GC exposure induced ∼10% increase of CD107a + CD4 + and CD8 + (UT and CAR) T cells, while three GC exposures drastically reduced the frequency of CD107a + T cells (p < 0.05, Fig. 3A) particularly in CD8 + UT in comparison to CD8 + CAR + T cells (p < 0.05). Overall, a single exposure slightly decreased the frequency of IFNγ + , TNF + and IL-2 + CD4 + and CD8 + T cells but no difference between UT and CD19 CAR T cells was observed (Additional file 1: Figure S3A). Contrarily, 3 GC exposures significantly decreased the frequency of cytokine producing CD4 + and CD8 + T cells (Fig. 3B). Repeated GC exposures strongly reduced the frequency of IFNγ + and TNF + UT T cells as compared to CD19 CAR T cells (p < 0.05, Fig. 3B). Accordingly, repeated exposure with GC significantly increased the frequency of nonfunctional (CD107-/IL-2-/TNF-/IFNγ-) CD4 + and CD8 + UT T cells (p < 0.05, Fig. 3C). UT and CAR T cells function was recovered by the supplement addition of GR antagonist RU-486 with a significant reduction of the frequency of nonfunctional (IL-2-/TNF-/IFNγ-) CD4 + and CD8 + T cells in this condition as compared to repeated Dx exposures only (p < 0.05, Additional file 1: Figure S3B).

Our results showed that the function of CD19 CAR T cells was less impacted than UT T cells by repeated GC exposures suggesting that CD19 CAR T cells may present more GC-resistant features than conventional T cells.

### Repeated CD19 CAR T cells activation with cognate antigen increased the negative impact of GC exposure.

In patients or in vivo models, efficient CAR T cell responses rely on serial killing of tumor cells. Here, over a 7-days period, CD19 CAR T cells were stimulated 1 or 3 times with CD19 + K562 target cells at a 1:1 E:T ratio before being exposed to low or high GC doses (Fig. 3D). A slight decrease of cytokine producing CD19 CAR T cells frequency, without significant impact was induced by GC exposure following a single stimulation with target cells (Additional file 1: Figure S3C). Yet, when assessing polyfunctionality, following a single stimulation with target cells, GC exposure diminished the proportion of quadruple (CD107 + /IL-2 + /TNF + /IFNγ +) and triple functional CD4 + CD19 CAR T cells (p < 0.05) and the proportion of nonfunctional (0 function: CD107-/IL-2-/TNF-/IFNγ-) CD4 + CD19 CAR T cells increased from 33.7% to 40.2% and 37.4% under Dx and MP exposure, respectively (Fig. 3E). After 3 stimulations with target cells, a single GC exposure greatly reduced CD19 CAR T cells frequencies of cytokines producing cells (Additional file 1: Figure S3D, p < 0.05). Conversely, repeated stimulations with target cells followed by GC exposure significantly from 30.7% to 51.7% with Dx and 50.7% with MP of the proportion of nonfunctional CD4 + CD19 CAR T cells (p < 0.05, Fig. 3E). While a similar trend was observed within CD8 + CD19 CAR T cells, the differences were lesser (Additional file 1: Figure S3E). Accordingly, GC increased significantly more the frequency of nonfunctional CD4 + CD19 CAR T cells after three repeated stimulations than after a single stimulation with target cells (p < 0.05, Fig. 3F).

Altogether, our data showed that GC exposure strongly reduced the function of activated (with cognate antigen) CD19 CAR T cells, and particularly repetitively stimulated CD4 + CD19 CAR T cells.

### The nature of the co-stimulatory domain (CD28 and 4-1BB) impacted differently the responses to GC exposure.

**Fig. 4:**
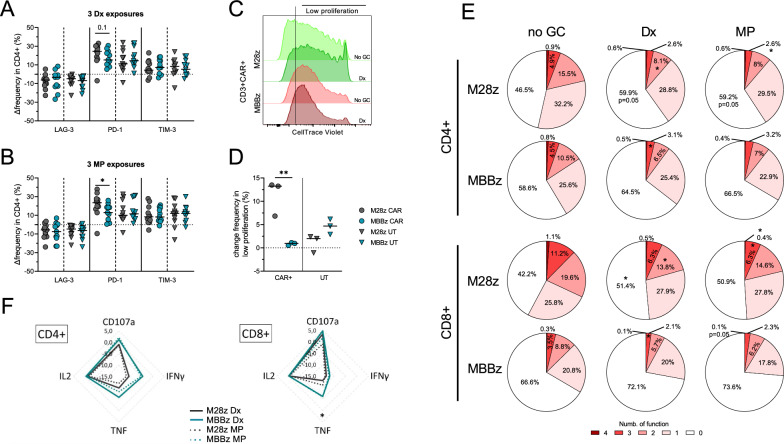
Impact of GCs exposure on M28z and MBBz CAR + T cells phenotype and functions. Relative surface expression of LAG-3, PD-1 and TIM-3 in CAR + and UT fractions of CD4 + M28z and MBBz T cells following 3 exposures with Dx (**A**) or MP (**B**). **C** Representative histogram of M28z and MBBz CAR T cells proliferation after 3 exposures with or without Dx over 6 days. **D** Relative frequency of low proliferative cells in the CAR + and UT fractions of M28z and MBBz T cells. **E** Pie chart showing the number of functions of CD4 + and CD8 + M28z and MBBz CAR T cells without GC exposure compared to Dx or MP exposure following 3 stimulations with MSLN + K562 tumor cells. Relative frequency of CD107a + (**F**), TNF (**G**), IFNγ + (**H**) and IL-2 + (**I**) CD4 + and CD8 + M28z and MBBz CAR T cells without GC exposure compared to Dx or MP exposure after MSLN + K562 tumor cells stimulation **A, B** n = 11, **D** n = 3, **F** n = 5 and **E** n = 5 donors. Mann–Whitney test and Student t test were used to compare the functions in M28z vs. MBBz. Friedman test with Dunn’s correction was used to compare multifunction in No GC vs. Dx vs. MP conditions. Medians are represented. * p < 0.05, ** p < 0.01

We next investigated how different CAR constructs were impacted by GC exposure. We used two CAR T cell constructs targeting MSLN that encode either a CD28 or 4-1BB co-stimulatory molecule (M28z and MBBz, respectively). Over a one week-period, MSLN CAR T cells were exposed 3 times to low and high concentration of GC before evaluating the phenotype of M28z and MBBz CAR T cells: CAR frequency and CD4/CD8 ratio was not impacted by the 3 GC exposures (Additional file 1: Figure S4A&B) but as observed with CD19 CAR T cells, GC exposure induced a significant decrease surface expression of LAG-3 and increase expression of PD-1 in M28z and MBBz CAR + and UT T cell fractions (Additional file 1: Figure S4C-E). Only PD-1 expression appeared to be more increased in the CD4 + subset of M28z CAR T cells as compared to MBBz CAR T cells, especially true during MP exposure (p < 0.05, Fig. 4A&B). This difference was not observed in the CD8 + M28z and MBBz CAR T cells (Additional file 1: Figure S4F&G). When analyzing the impact of GC on the proliferative capacity, we observed that M28z CAR T cells appeared to be the most impacted as shown by a higher increase (median ∼12%, p < 0.01) of the low proliferative cell fraction (Fig. 4C&D).

The cytotoxic capacity of the MSLN CAR T cells was then evaluated following three stimulations with MSLN + K562 at a 1:1 E:T ratio before being exposed to a single low or high dose of GC. GC exposures induced a decreased frequency of IFNγ + and TNF + CD4 + and CD8 + T cells in MSLN CAR T cells (Additional file 1: Figure S4H&I). Accordingly, Dx or MP exposure decreased the polyfunctionality (Fig. 4E): Although, without GC exposure, MBBz CAR T cells appeared to be less functional than M28z CAR T cells (58.6% vs. 46.5% of CD4 + and 66.6% vs. 42.2% of CD8 + , respectively), M28z CAR T cells were more impacted than MBBz CAR T cells with a significant increase of nonfunctional CD4 + (Dx and MP, p = 0.05) and CD8 + T cells (Dx, p < 0.05). Accordingly, polyfunctional CD4 + and CD8 + T cells were reduced upon GC exposure, especially in M28z CAR T cells (p < 0.05, 2–4 functions, Fig. 4E). When analyzing frequency changes in degranulation and cytokine production upon GC exposure, M28z CAR T cells tended to be more impacted by GC (Fig. 4F), as highlighted by a stronger decrease in TNF + CD8 + M28z as compared to MBBz CAR T cells (p = 0.05 Fig. 4F).

Altogether, GC impacted MSLN CAR T cells differently depending on the co-stimulatory domain (CD28 or 4-1BB). MSLN CAR T cells encoding CD28 exhibited a stronger increase of PD-1 expression, an observable lower proliferative capacity, and a more reduced functionality than MSLN CAR T cells encompassing the 4-1BB co-stimulatory domain.

### CAR T cells reduced antitumor activity and slowed recovery upon repeated GC exposures.

**Fig. 5:**
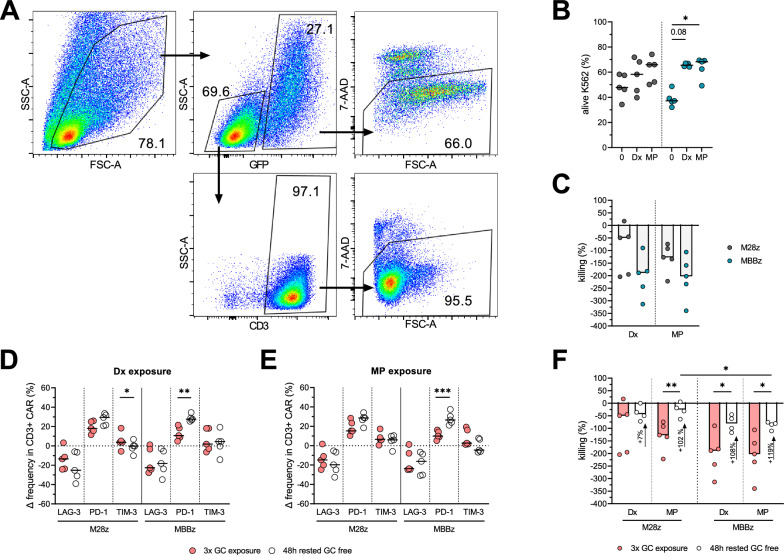
Recovery of M28z and MBBz CAR T cells functions after GC exposure. A. Representative flow cytometry analysis used for FACS-based killing assay. **B** Frequency of viable GFP + MSLN + K562 target cells after 24h incubation with GC-exposed M28z or MBBz CAR T cells. **C** Frequency of killing reduction of M28z and MBBz induced by 3 exposures with Dx or MP. Relative frequency of LAG-3, PD-1 and TIM-3 in M28z and MBBz CAR T cells after 3 exposures with Dx (**D**) or MP (**E**) and 48h rest in a GC-free medium. **F** Frequency of killing reduction of M28z and MBBz CAR T cells induced by 3 exposures with Dx or MP and 48h rest in a GC-free medium. n = 5 donors. Friedman test with Dunn’s correction was used to compare K562 viability in No GC vs. Dx vs. MP conditions. Student t test was used to compare surface marker expression in GC-exposed vs. GC-rested conditions. Medians are represented. * p < 0.05, **p < 0.01, *** p < 0.001

We next evaluated CD28 and 4-1BB MSLN CAR T cells antitumor potency against K562 tumor cells expressing GFP and MSLN after 3 GC exposures (10 µg/ml Dx and 50 µg/ml MP) over a one-week period using a flow cytometry based killing assay (Fig. 5A and Additional file 1: Figure S5A). Higher frequency of viable MSLN + K562 tumor cells was observed when co-incubated with MBBz CAR T cells previously exposed to GCs (Fig. 5B). Accordingly, normalized killing potency for MBBz CAR T cells was reduced up to 200% upon GC repeated exposures. No difference in killing inhibition was observed between Dx and MP (Fig. 5C). GC-exposed MSLN CAR T cells were then transferred to a GC-free medium for 48h to investigate their recovery ability. Unexpectedly, after a 48h GC-free rest, no reduction on the changes induced by GC exposures was observed, instead, PD-1 expression in M28z and MBBz CAR T cells was further increased (p < 0.05, Fig. 5D&E, Additional file 1: Figure S5B-E). Despite this noticeable amplified phenotypical difference after GC-free rest, the killing capacity appeared to recover from GC exposure, yet not entirely (p < 0.05, Fig. 5F). M28z CAR T cells seemed to have almost entirely recovered from MP exposure (median killing frequency reduction < 50%). Furthermore, MBBz CAR T cells killing capacity significantly improved (+ 100%, p < 0.05, Fig. 5F).

Overall, repeated GC exposures significantly reduced the killing capacity of MSLN CAR T cells especially MBBz CAR T cells. M28z and MBBz CAR T cells were able to partially recover their killing capacity within 48h hours despite exhibiting an affected phenotype. No apparent difference between Dx and MP in the killing and recovery impact on MSLN CAR T cells was observed.

## Discussion

Little is known about the interaction between new immunotherapies and “old” standard treatments. Due to their strong immunosuppressive potency, GCs are used carefully to manage CAR-treatment-related side effects without abolishing the CAR T cells anti-tumor activity and increasing the risk of relapse [16]: Several retrospective analyses of the effect of GCs in patients treated with CAR T cells therapy have described that high, repeated and prolonged doses of GC reduce progression-free survival, but no general consensus has been drawn concerning the real impact of GC therapy on the various existing CAR T cell treatments [11]: if T cells and CAR T cells respond similarly to GC, or if different CAR T cell products (e.g. different co-stimulatory domains) differ in response to GC exposure.

To our knowledge, only two in vitro reports have described the deleterious effect of GCs on CAR T cells: They reported that GCs, in a dose-dependent manner, to reduce CD19-CAR T cells proliferation and their killing activity against different tumor cells [17]. Our results differed from this report describing that Dx was more potent than MP as we used the 2 GCs at dose equivalence (1mg Dx ≈5mg MP [14]), hence, we did not distinguish differences between the effect of Dx and MP on CAR T cells. The second study also demonstrated the dose-dependent effect of Dx in reducing the efficacy to eliminate cancer cells of IL13Rα2 CAR T cells [18]. Yet, no study reported phenotypical or functional comparison between T cells and T cells expressing different CAR constructs.

First, we showed that the highest GR expression level was mainly found in the naïve and TCM CAR T cells while more differentiated cells (that represent most T cells in the CAR products [19]) expressed significantly less GR. Since TCM cells have high self-renewal capacity and may play an important in providing long-term anti-tumor immunity [20], the increased negative impact of GC on this subset may dampen long term CAR T cells efficacy. A report in a mouse model showed a significant reduction in proliferation and IFNγ production by naïve CD8 + T cells as compared to activated CD8 + T cells [21]. Moreover, transient Dx treatment induced apoptosis of naïve and memory CD8 + T cells but not on herpes virus-specific CD8 + T cells demonstrating variation in GC sensitivity according to the T cell subsets analyzed [9].

Even though UT and CAR + T cells displayed altogether a similar phenotype (expression of differentiation markers and GR) our data showed that CD19 CAR + T cells were less negatively affected (as seen by expression of ICMs and effector functions) by GC exposure than UT T cells. CAR expression by T cells is known to induce tonic signaling, a spontaneous activation in the absence of activation with cognate antigen [22]. CAR T cells tonic signaling may underline the increased resistance of CAR T cells to GC exposure. Whether CAR T cells with high and low tonic signaling respond differently to GC exposure would be of interest. Additionally, by suppressing fatty acid metabolism essential for memory T cells, GCs decreased memory CD8 + T cells with low TCR affinity due to their lower phosphorylation of GR [23]. Since a recent study showed enhanced functionality of low-affinity in comparison to high-affinity CAR T cells [24], investigating whether GCs select CAR T cells depending on the CAR affinity may be pertinent.

We showed that GC exposure after prior activation of CD19 CAR T cells with cognate antigen (and particularly multiple stimulations), that mirrors the in vivo setting, induced a decrease in CD19 CAR T cells effector functions, in particularly CD4 + T cells. The differential effect of GCs on CD4 + and CD8 + T cells may be attributed to variations in receptor expression (and its phosphorylation), sensitivity to GC signaling or functional and metabolic pathways in these two T cell subsets [25, 26].

Due to the different activation intensity and downstream signaling pathway, the nature of the CAR co-stimulatory domain plays a crucial role in CAR T cells functions [27], therefore we compared MSLN directed CAR T cells that encompassed either the CD28 or the 4-1BB domain and showed that GCs impacted more negatively M28z CAR T cells than MBBz CAR T cells with an increased PD-1 frequency and reduced function. Interestingly, it has been shown that T cell function can be suppressed through PD-1 by inactivation CD28 signaling [28, 29] and that GCs attenuated the CD28 pathway and reduce cytokine production such as IL-2 [30, 31]. Therefore, those mechanisms could explain the increased sensitivity to GCs of CAR T cells encompassing a CD28 co-stimulation domain as compared to MBBz.

We assessed the effect of GCs on CD19 and MSLN CAR T cells yet, the impact of GCs is context and dose-dependent and influences many cell subsets [32]; additional studies are therefore needed, for example on macrophages (paramount to CRS/ICANS development), to evaluate a more complete picture on the impact of GCs in the context of CAR T cell therapy [3]. Furthermore, while we described the difference of GR between memory subsets of T cells, we only assessed the short-term GC impact on CAR T cells phenotype and functions. Early studies on immune checkpoints blockades (ICB) described no negative impact of GCs administration on patient outcome, but a long-term (> 6years) retrospective study showed a reduced overall survival in melanoma patients who received GCs to treat adverse events induced by ICB [33]. It would therefore be interesting to investigate the long-term effect of GCs on memory CAR T cells, which are crucial for long-term immunity against relapse.

## Conclusion

In conclusion, the current study showed that GCs impaired more strongly untransduced T cells than CAR T cells and CD4 + T cells. Furthermore, CAR T cells encoding CD28 and 4-1BB co-stimulatory domains responded differently to GCs exposure.

### Supplementary Information

**Additional file 1: Figure S1. **UT T cells phenotype. A Representative plot of the differentiation subsets defined by CCR7 and CD45RA markers. B Memory phenotype of CAR + and untransduced (UT) fractions of CD3 + T cells in CD19, M28z and MBBz CAR T cell products.GR expression (MFI) in the naïve and TCM memory subsets of (C) UT (from CD19 CAR cell product) CD4 + and CD8 + T cells; (D) of the UT (from M28z and MBBz CAR cell products) CD4 + (left) and CD8 + (right) T cells. n = 5 donors for CD19 CAR T cells and n = 4 donors for M28z and MBBz CAR T cells. Student t test was used to compare GR in different subsets. Medians are represented. *p < 0.05, **p < 0.01. **Figure S2. **Impact of GC exposure on UT T cells phenotype. Frequency of CD19 CAR T cells (A) and CD4/CD8 ratio in CD19 CAR T cells (**B**) overtime after Dx (green) or MP (blue) exposure. C. Representative plot of LAG-3, PD-1 and TIM-3 expression without GC exposure or with 10µg/ml Dx. **D.** Frequency of LAG-3, PD-1, and TIM-3 in CD19 CAR + T cells without or after 1 and 3 exposures with Dx or MP. Relative surface expression of LAG-3, PD-1, andTIM-3 in CAR + and UT CD4 + (E) and CD8 + (F) T cells after 3 exposures with Dx (left) or MP (right). G Frequency of PD-1 in CD4 + and CD8 + CD19 CAR + T cells without or after 3 exposures with Dx or after 3 exposures with Dx and RU-486 (10^−5^M). H Relative expression of LAG-3, PD-1, and TIM-3 in CD4 + and CD8 + UT T cells after 3 exposures of Dx (left) or MP (right). n = 6 donors. Friedman test with Dunn’s correction was used to compare marker’s expression between 3 conditions. Wilcoxon matched-pairs signed rank test was used to compare marker’s expression in CAR + vs. UT or CD4 + vs. CD8 + T cells. Medians are represented. * p < 0.05, ** p < 0.01, *** p < 0.001. **Figure S3. **Impact of GCs exposure on CD19 CAR + T cells effector functions. A. Relative frequency of IFNγ + , TNF + or IL2 + CD4 + or CD8 + CD19 CAR and UT after a single exposure with Dx (top) or MP (bottom) after PMA/ionomycin stimulation. B. Frequency of CD107a- IFNγ- TNF- IL2- CD4 + and CD8 + CAR (circle) and UT (square) T cells after 3 exposures with Dx or Dx + RU-486 after PMA/ionomycin stimulation. Frequency of CD107a + , IFNγ + , TNF and IL-2 + CAR CD4 + and CD8 + T cells without GC exposure (0) or 1 exposure with low or high doses of GC (0.1 µg/ml and 10 µg/ml Dx and 0.5µg/ml, 50 µg/ml, and 100 µg/ml MP) following a single (C) or 3 (D) stimulations with CD19 + K562 tumor cells. E. Pie chart showing the number of functions of CD8 + CAR T cells without GC exposure compared to Dx or MP exposure following a single (n = 8 donors) or 3 stimulations (n = 5 donors) with CD19 + K562 tumor cells. Two-way ANOVA with Sidak’s correction was used to compare CD107a + , IFNγ + , TNF and IL-2 + cells frequency at different doses of Dx and MP. Friedman test with Dunn’s correction was used to compare multifunction between the 3 different conditions. Medians are represented. * p < 0.05, ** p < 0.01, *** p < 0.001. **Figure S4**. Impact of GCs exposure on M28z and MBBz CAR + T cells phenotype and functions. Frequency of M28z and MBBz CAR T cells (A) and CD4/CD8 ratio in CAR T cells (B) without GC exposure (0) or after 3 exposures with low and high concentration of Dx or MP. Frequency of LAG-3 (C), PD-1 (D) and TIM-3 (E) in M28z and MBBz CAR T cells without GC exposure (0) or after 3 exposures with low and high concentration of Dx or MP. Relative surface expression of LAG-3, PD-1 and TIM-3 in CAR + and UT fractions of CD8 + M28z and MBBz T cells following 3 exposures with Dx (F) or MP (G). Frequency of CD107a + , IFNγ + , TNF and IL-2 + CD4 + (H) and CD8 + (I) M28z and MBBz CAR T cells without GC exposure (0) compared to Dx or MP exposure following 3 stimulations with MSLN + K562 tumor cells. A-E n = 6, F&G n = 11 and H&I n = 5 donors. Two-way ANNOVA with Sidak’s correction was used to compare CD107a + , IFNγ + , TNF and IL-2 + cells frequency at different doses of Dx and MP. Mann–Whitney test was used to the surface expressions and functions in M28z vs. MBBz. Medians are represented. * p < 0.05, ** p < 0.01, *** p < 0.001. **Figure S5. **M28z and MBBz CD4 + and CD8 + CAR T cells phenotype after GC removal. A Representative histograms of GFP and MSLN expression and co-expression on K562 target cells as compared to control K562 (GFP-MSLN-). Relative frequency of LAG-3, PD-1 and TIM-3 in M28z and MBBz CD4 + (B&D) and CD8 + (C&E) CAR T cells after 3 exposures with Dx (B-C) or MP (D-E) and 48h rest in a GC-free medium. n = 5 donors. Student t test was used to compare surface marker expression in GC-exposed vs. GC-rested conditions. * p < 0.05, ** p < 0.01, *** p < 0.001.

**Additional file 2.** Additional data.

## Data Availability

Data generated and used and/or analyzed during are available from the corresponding author on reasonable request.

## Abbreviations

ALL: Acute lymphoblastic leukemia
CAR: Chimeric antigen receptor
CRS: Cytokine release syndrome
Dx: Dexamethasone
GC: Glucocorticoid
GR: Glucocorticoid receptor
ICANS: Immune effector cell-associated neurotoxicity syndrome
ICB: Immune checkpoints blockades
ICM: Immune checkpoint markers
MP: Methylprednisolone
MSLN: Mesothelin
TCM: T central memory
TEM: T effector memory phenotype
UT: Untransduced
