# Supplementary figures and images for: CAR T cells and T cells phenotype and function are impacted by glucocorticoid exposure with different magnitude
Source: J Transl Med. 2024 Mar 12;22:273. doi: 10.1186/s12967-024-05063-4 (PMC10935894; doi:10.1186/s12967-024-05063-4)

Suppl Figure 1

**A**

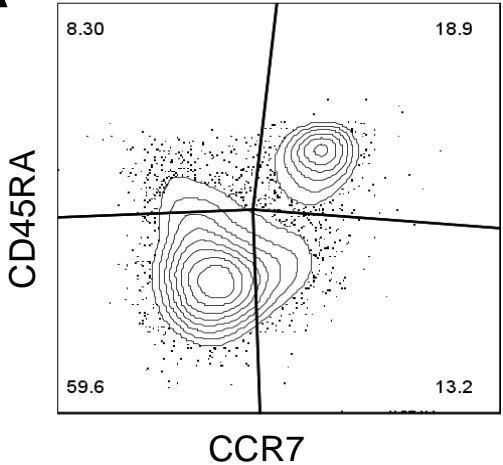

**B**

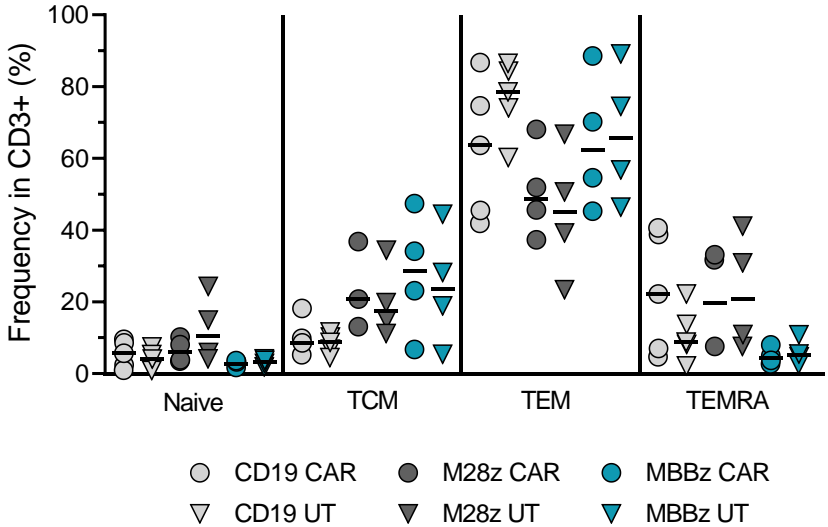

**C**

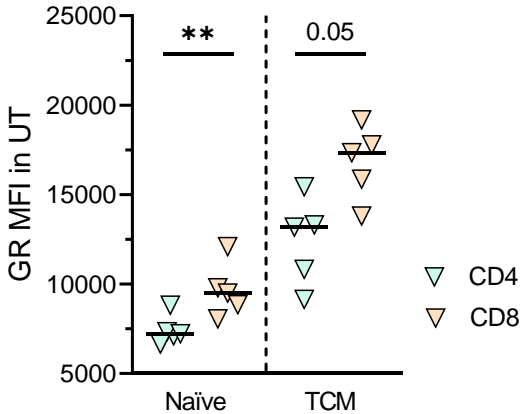

**D**

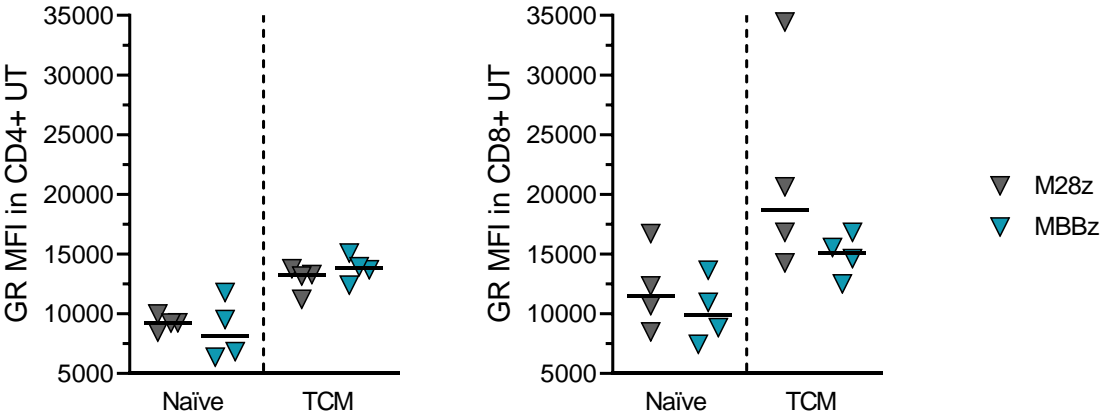

Suppl Figure 2

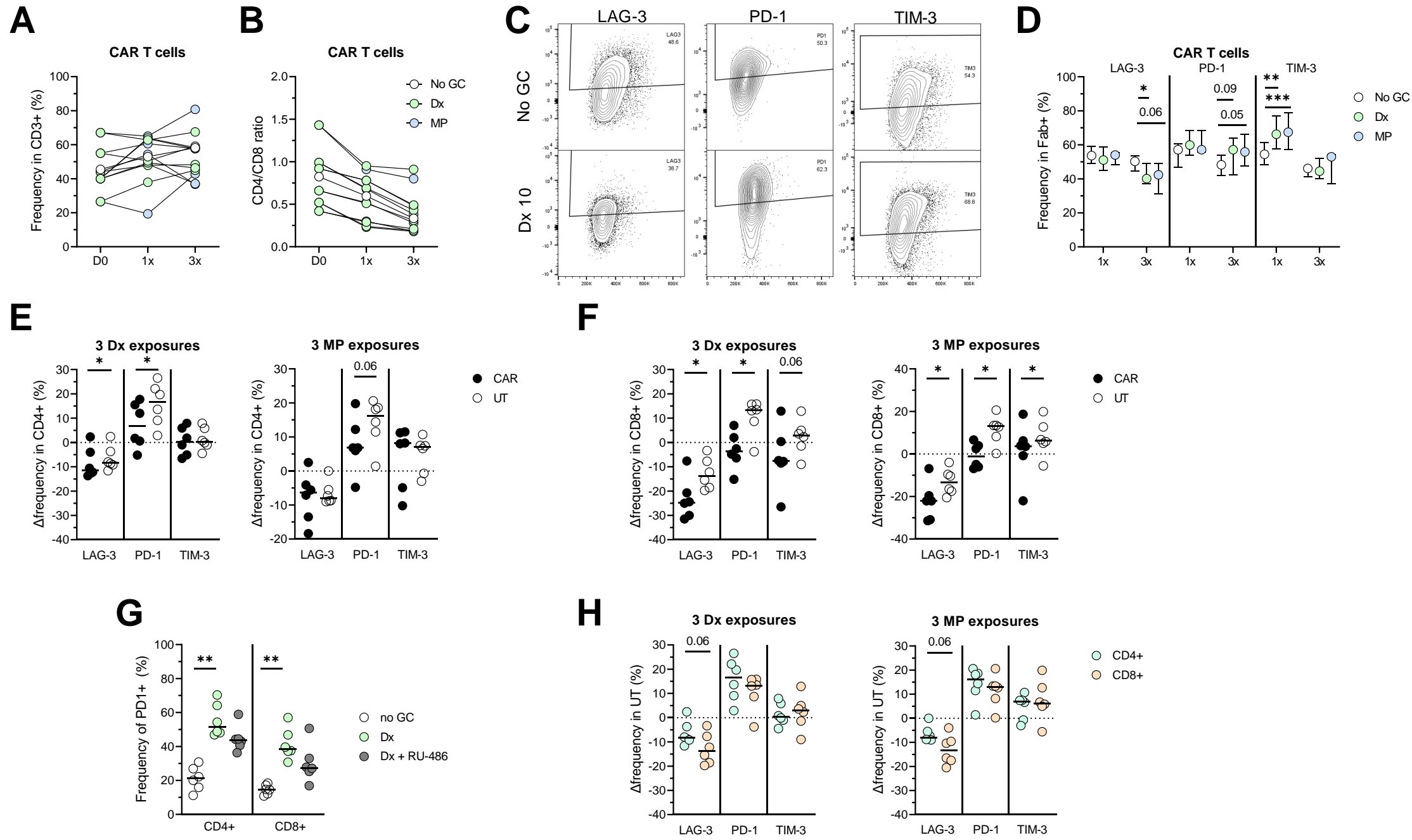

Suppl Figure 3

**A**

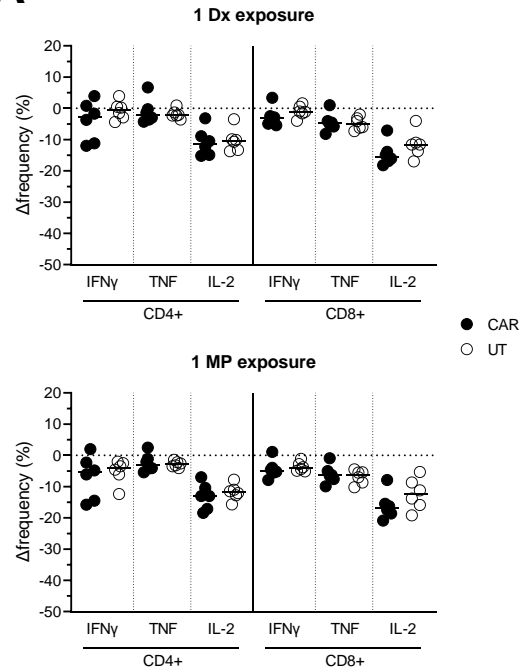

**B**

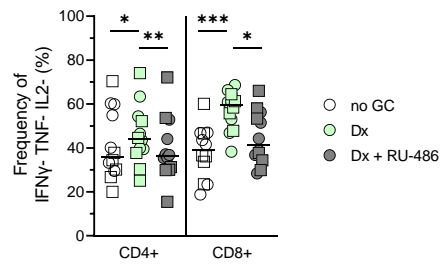

**C**

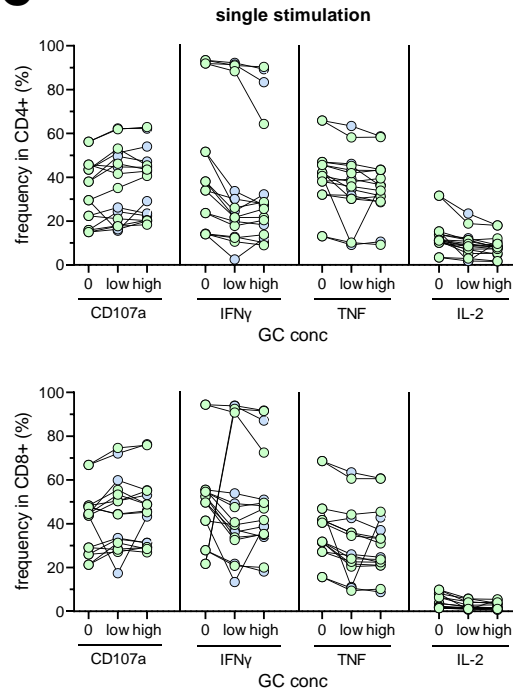

**D**

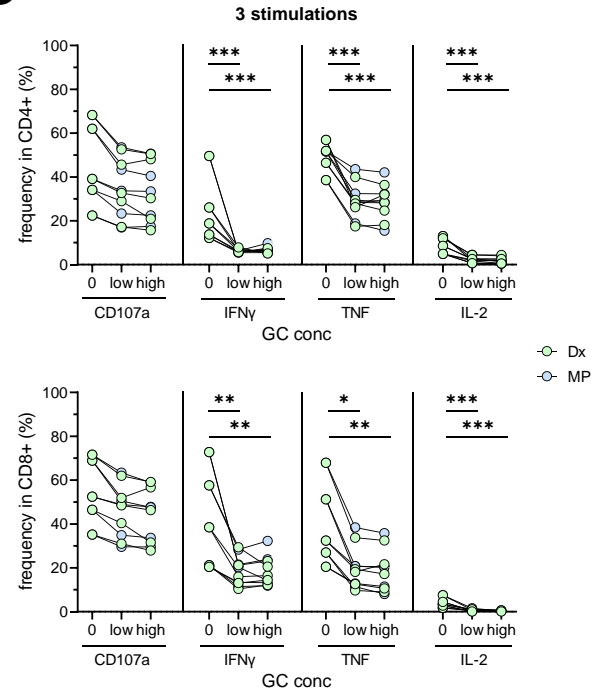

● Dx  
● MP

**E**

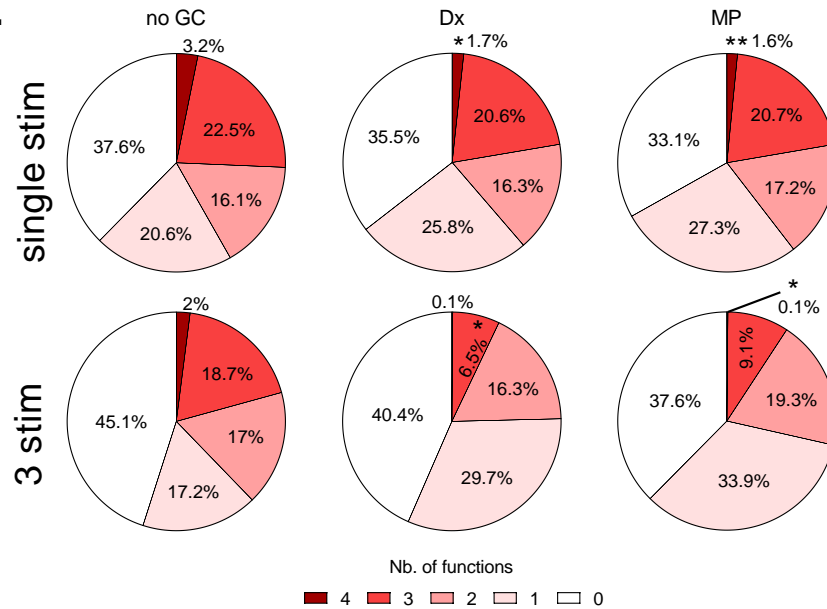

Suppl Figure 4

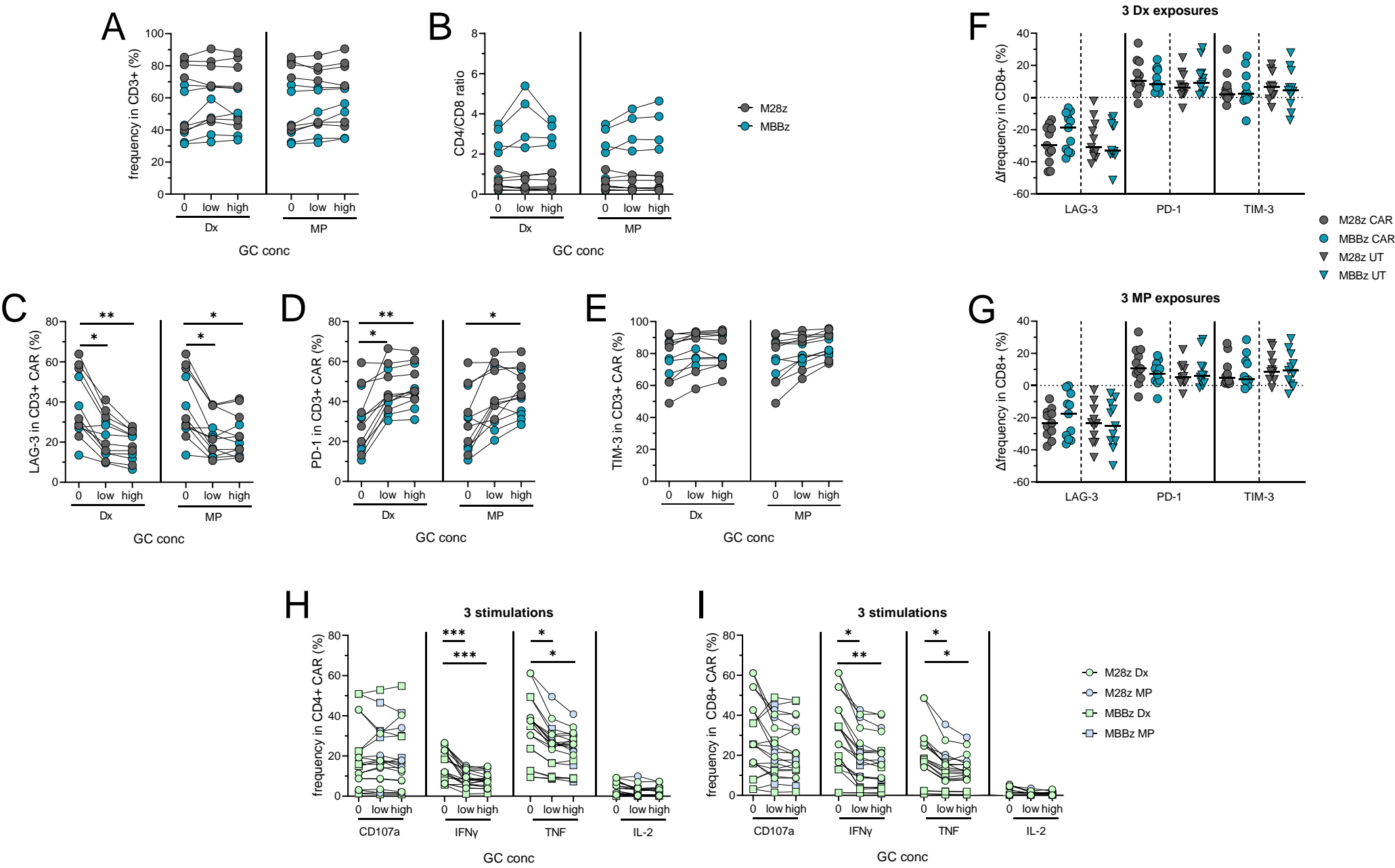

Suppl Figure 5

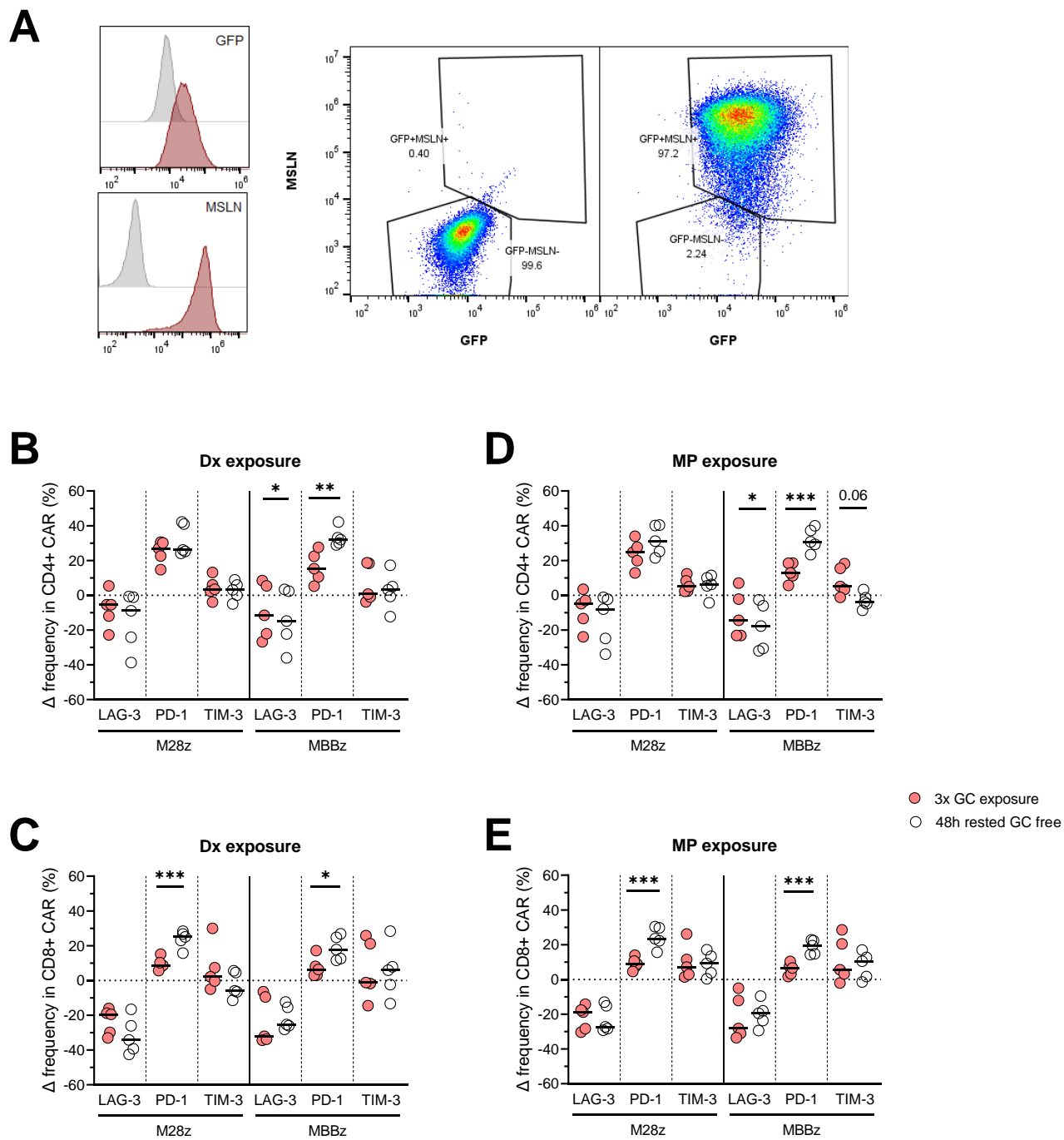

Supplement: Supplementary file 1 — Additional file 1: Figure S1. UT T cells phenotype. A Representative plot of the differentiation subsets defined by CCR7 and CD45RA markers. B Memory phenotype of CAR + and untransduced (UT) fractions of CD3 + T cells in CD19, M28z and MBBz CAR T cell products.GR expression (MFI) in the naïve and TCM memory subsets of (C) UT (from CD19 CAR cell product) CD4 + and CD8 + T cells; (D) of the UT (from M28z and MBBz CAR cell products) CD4 + (left) and CD8 + (right) T cells. n = 5 donors for CD19 CAR T cells and n = 4 donors for M28z and MBBz CAR T cells. Student t test was used to compare GR in different subsets. Medians are represented. *p < 0.05, **p < 0.01. Figure S2. Impact of GC exposure on UT T cells phenotype. Frequency of CD19 CAR T cells (A) and CD4/CD8 ratio in CD19 CAR T cells (B) overtime after Dx (green) or MP (blue) exposure. C. Representative plot of LAG-3, PD-1 and TIM-3 expression without GC exposure or with 10µg/ml Dx. D. Frequency of LAG-3, PD-1, and TIM-3 in CD19 CAR + T cells without or after 1 and 3 exposures with Dx or MP. Relative surface expression of LAG-3, PD-1, andTIM-3 in CAR + and UT CD4 + (E) and CD8 + (F) T cells after 3 exposures with Dx (left) or MP (right). G Frequency of PD-1 in CD4 + and CD8 + CD19 CAR + T cells without or after 3 exposures with Dx or after 3 exposures with Dx and RU-486 (10−5M). H Relative expression of LAG-3, PD-1, and TIM-3 in CD4 + and CD8 + UT T cells after 3 exposures of Dx (left) or MP (right). n = 6 donors. Friedman test with Dunn’s correction was used to compare marker’s expression between 3 conditions. Wilcoxon matched-pairs signed rank test was used to compare marker’s expression in CAR + vs. UT or CD4 + vs. CD8 + T cells. Medians are represented. * p < 0.05, ** p < 0.01, *** p < 0.001. Figure S3. Impact of GCs exposure on CD19 CAR + T cells effector functions. A. Relative frequency of IFNγ + , TNF + or IL2 + CD4 + or CD8 + CD19 CAR and UT after a single exposure with Dx (top) or MP (bottom) after PMA/ionom [file 12967_2024_5063_MOESM1_ESM.pdf]
