# Supplementary material for: CAR T cells and T cells phenotype and function are impacted by glucocorticoid exposure with different magnitude
Source: J Transl Med. 2024 Mar 12;22:273. doi: 10.1186/s12967-024-05063-4 (PMC10935894; doi:10.1186/s12967-024-05063-4)
Supplement: Supplementary file 2 — Additional file 2. Additional data. [file 12967_2024_5063_MOESM2_ESM.pdf]

CAR T cells and T cells phenotype and function are impacted by glucocorticoid exposure with different magnitude.

Thomas Poiret[1], Sara Vikberg[1], Esther Schoutrop[1], Jonas Mattsson[1,2,*], Isabelle Magalhaes[1,3,*]

Supplementary information

Materials &Methods

CAR T cells and target cells culture. Retroviral supernatant production was performed according as previously reported [1]. CAR transduction efficiency was determined by the surface expression using an anti-mouse Fab (to detect CD19 CAR) and anti-EGFRt/Cetuximab (to detect MSLN CAR). Until stated differently, CAR T cells were cultured in AIM-V medium supplemented with 5% human serum and 300 IU/mL IL-2. K562 cells were transduced with retrovirus coding human MSLN variant 1 (SFG vector, provided by Prof. M. Sadelain, MSKCC). CD19+ K562, NGFR+ K562 (kindly provided by Dr Steven Feldman, National Cancer Institute, Bethesda) and MSLN+ K562 (expressing or not GFP) were cultured in RPMI (GE Healthcare Life Sciences, HyClone) supplemented with 10% fetal bovine serum (FBS, ThermoFisher Scientific, HyClone), 100 U/mL of Penicillin and 100 µg/mL Streptomycin (ThermoFisher Scientific, HyClone).

Flow cytometry analysis. To analyze the GC Receptor (GR) expression level, cell viability was assessed using LIVE/DEAD Fixable Aqua Dead Cell Stain (Molecular Probes). CAR T cells targeting MSLN or CD19 were stained with a primary biotinylated anti-EGFRt/Cetuximab antibody (R&D Systems) or a primary biotinylated anti-mouse Fab (Jackson ImmunoResearch Lab) then blocked with mouse gamma globulin (Jackson ImmunoResearch Lab) as described previously [2]. Cells were washed twice prior to staining with Streptavidin-APC (Biolegend). Other cell surface markers included PE/Cy7-CD3 (BD), APC/Cy7-CD8 (BD), AF700-CD4 (BD), BV785-CD45RA (Biolegend), BV421-CCR7 (Biolegend) antibodies. Foxp3 / Transcription Factor Staining Buffer Set (eBioscience™) was used with the FITC-GR (Santa Cruz Biotech) antibody for intra-cellular/-nuclear staining and FITC-mouse IgG1 (Biolegend) used as isotype control. Median fluorescence intensity (MFI) was used to evaluate the GR expression level.

Immunophenotyping was performed after a single or 3 exposures with GCs over a period of 6 days. Following anti-Fab or -EGFRt staining as described above, cells were stained with: FITC-PD-1 (BD), Streptavidin-PE (BioLegend), PE/Cy7-CD3 (BD), APC/Cy7-CD8 (BD), BV510-CD4 (BioLegend), BV650-LAG3 (BioLegend) and APC- TIM-3 (Miltenyi Biotec) or BV785-TIM-3 (BioLegend) antibodies, followed by 7-AAD staining.

Intracellular staining was performed as described previously to assess the specific production of IFNγ, TNF, IL-2 and degranulation by CD107a after stimulation with phorbol myristate

acetate (PMA, Sigma-Aldrich) and ionomycin (Sigma-Aldrich) or K562 target cells expressing CD19 or MSLN at 1:1 E:T ratio [3].

Proliferation was assessed after 3 exposures with different GC doses over a period of 6 days using the CellTrace Violet dye (Molecular Probes) at a 5 µM concentration.

Killing readout was assessed by flow cytometry analysis using PE/Cy7-CD3, BV510-CD4 and APC/Cy7CD8 antibodies in combination with GFP (K562 target cells) and 7-AAD.

Cells were acquired on a CytoFlex (Beckman Coulter) flow cytometer and data were analysed using FlowJo™ v10.8 Software (BD Life Sciences). The number of functions (from 0 to 4) and co-expression of markers (LAG-3, PD-1 and TIM-3) were assessed by the Boolean gate analysis. The number of functions was graded from 0 (dysfunctional T cells: CD107a- IFNγ -TNF- IL2-) to 4 (CD107a+ IFNγ+ TNF+ IL-2+).

References

1. Alnabhan R, Gaballa A, Mork LM, Mattsson J, Uhlin M, Magalhaes I. Media evaluation for production and expansion of anti-CD19 chimeric antigen receptor T cells. *Cytotherapy*. 2018;20(7):941-951.
2. Schoutrop E, El-Serafi I, Poiret T, et al. Mesothelin-Specific CAR T Cells Target Ovarian Cancer. *Cancer Res*. 2021;81(11):3022-3035.
3. Magalhaes I, Kalland I, Kochenderfer JN, Osterborg A, Uhlin M, Mattsson J. CD19 Chimeric Antigen Receptor T Cells From Patients With Chronic Lymphocytic Leukemia Display an Elevated IFN-gamma Production Profile. *J Immunother*. 2018;41(2):73-83.
